# Neoadjuvant chemoimmunotherapy in patients with locally advanced squamous head and neck cancer: A retrospective study

**DOI:** 10.3389/fonc.2025.1576800

**Published:** 2025-10-14

**Authors:** Qiu-Yi Huang, Ling-Feng Xu, Yuan Wu, Jian Chen

**Affiliations:** ^1^ Department of Head and Neck Surgery, Hubei Cancer Hospital, Tongji Medical College, Huazhong University of Science and Technology, Wuhan, China; ^2^ Department of Radiation Oncology, Hubei Cancer Hospital, Tongji Medical College, Huazhong University of Science and Technology, Wuhan, China

**Keywords:** head and neck squamous cell carcinoma, neoadjuvant therapy, immunotherapy, chemotherapy, pathological response

## Abstract

**Background:**

The conventional approach for the treatment of locally advanced head and neck squamous cell carcinoma (HNSCC) entails the combination of surgery with radiotherapy or chemoradiotherapy. However, the survival rate of patients has not improved. This is frequently attributed to the strong invasive and metastatic capabilities of the tumor, which makes it prone to recurrence. In recent years, neoadjuvant chemoimmunotherapy has emerged as a focal point of research. This is primarily due to its remarkable enhancement of the pathological response rate and patient survival. The objective of this study was to conduct a retrospective analysis to evaluate the safety and efficacy of neoadjuvant chemoimmunotherapy in locally advanced HNSCC.

**Methods:**

The clinical data of 82 patients with HNSCC, who underwent surgery subsequent to neoadjuvant chemoimmunotherapy during the period from January 1, 2019, to May 31, 2024, were retrospectively incorporated in this study. Analyses were conducted on the pathological response rate, survival data, and adverse events associated with the treatment.

**Results:**

This study enrolled 82 patients in total. The oral cavity was the site of malignancies in 41, 50.0% of the cases. Nearly half of the patients (32, 39.0%) were treated with two cycles of neoadjuvant chemoimmunotherapy, while the remaining patients received three or more cycles. 78 patients (95.1%) achieved R0 resection and 65 patients (79.3%) achieved objective response rate (ORR). The pathological complete response (pCR) rate was 25.6% [95% confidence interval (CI), 15.3%–35.1%], and the major pathological response (MPR) rate was 41.5% (95% CI, 30.4%-52.0%). All patients demonstrated good tolerance of neoadjuvant therapy, with a grade 3/4 treatment-related adverse event rate of 14.6%. After a median follow-up of 16 (3–48) months, the 1-year disease-free survival rate was 80.5% (95%CI: 72.4%- 88.6%) and the 1-year overall survival rate was 93.7% (95%CI: 88.7%-99.14%).

**Conclusions:**

Neoadjuvant chemoimmunotherapy significantly improves the pathological response rate and R0 resection rate in patients with locally advanced HNSCC with a low incidence of treatment-related adverse events, and our findings suggest its potential as a treatment strategy for locally advanced HNSCC.

## 1 Introduction

Approximately 500,000 people die of head and neck squamous cell carcinoma (HNSCC) every year, which ranks as the sixth most prevalent malignant tumor worldwide (1). These tumors are commonly found in the oral cavity, pharynx, larynx and other parts, and their incidence is increasing year by year due to high-risk factors such as smoking, alcohol consumption and human papillomavirus (HPV) infection (2). About 60% of patients with HNSCC are diagnosed with locally progressed illness, which indicates that the tumor may have spread to nearby tissues or lymph nodes, despite advancements in medical technology. The 5-year survival rate is less than 50%, and the risk of distant metastasis and local recurrence remains high even if these patients undergo complete treatment that includes chemotherapy, radiation therapy, and surgery (3, 4). Therefore, the need to develop more effective treatment methods is very pressing.

Neoadjuvant therapy is currently becoming more popular as a preoperative treatment option. By decreasing tumor size, it seeks to lower the rate of postoperative recurrence and enhance patients’ overall survival prognosis. Although preoperative neoadjuvant chemotherapy has been extensively studied recently, it has not been found to increase the survival of patients with locally advanced HNSCC, according to the findings of multiple phase III clinical studies (5, 6). Researchers have started looking into the possible use of immunotherapy in neoadjuvant therapy due to the success of clinical trials like KEYNOTE-048 in the treatment of recurrent or metastatic HNSCC (7–9). Neoadjuvant immunotherapy is thought to induce clonal expansion of tissue-resident memory T cells in the primary focus and enhance the immune response in the tumor microenvironment, thus potentially improving patient survival (10, 11). Neoadjuvant immunotherapy has improved the survival of patients with locally advanced HNSCC, according to multiple follow-up studies. However, the efficacy of single-agent immunotherapy is low, with the major pathological response (MPR) of only 5.9-15% (12–14). This implies that a patient’s immune system could not be adequately stimulated by a single immunotherapy to combat the tumor successfully.

To further enhance pathological response rates, researchers have shifted focus to ​​combination strategies integrating neoadjuvant immunotherapy with chemotherapy. Several studies have shown that neoadjuvant chemoimmunotherapy not only successfully reduces tumor volume, but also improves local lesion control, reduces the risk of distant metastasis, and improves the long-term survival of patients (15–19). Chemotherapy can directly kill tumor cells, promote antigen release, and activate the inflammatory tumor microenvironment. These supporting antigen cross-presentation and anti-tumor immune response are the theoretical basis of neoadjuvant chemoimmunotherapy (20). However, current clinical trials on neoadjuvant chemoimmunotherapy still lack sufficient evidence in terms of confirming long-term survival benefits for patients. At the same time, the limited sample size makes it difficult to fully assess their true effects in the clinical setting.

Since 2019, our institution has implemented a neoadjuvant chemoimmunotherapy protocol for locally advanced HNSCC patients, employing a regimen comprising paclitaxel, gemcitabine, platinum-based agents, fluorouracil, and programmed cell death protein 1 (PD-1) inhibitors. During this treatment, the physician consults with the patient to select the most appropriate immunotherapy regimen based on the patient’s specific situation. In this study, we analyzed patients with locally advanced HNSCC who received neoadjuvant chemoimmunotherapy and underwent surgical resection at our institution to evaluate the effectiveness and feasibility of this treatment modality, to provide theoretical support for the optimization of treatment strategies.

## 2 Materials and methods

Patient selection: This retrospective cohort study analyzed patients with locally advanced HNSCC who underwent neoadjuvant chemoimmunotherapy and radical surgery between January 2019 and May 2024 at Hubei Provincial Cancer Hospital. Eligible patients were required to meet the following criteria: age between 18–75 years with a performance status (PS) score of 0-1; histologically confirmed squamous carcinoma originating in the oral cavity, oropharynx, larynx, or hypopharynx; clinical stage III-IVB according to the AJCC 8th edition criteria; radiologically resectable lesions without distant metastases confirmed by multidisciplinary evaluation; and completion of at least two cycles of neoadjuvant chemoimmunotherapy before surgery. The study followed the Declaration of Helsinki and was approved by the Medical Ethics Committee of Hubei Provincial Cancer Hospital, Tongji Medical College, Huazhong University of Science and Technology (Ethical Review No. *LLHBCH202YN-078*), and no informed consent was required from the patients as the study was observational and non-interventional.

Data collection: Clinical TNM staging of patients with HNSCC was performed according to the AJCC 8th edition, and patient demographic and clinical information was collected, including gender, age at diagnosis, history of smoking and alcohol consumption, tumor primary, HPV 16 status (oropharyngeal carcinoma), clinical stage, neoadjuvant therapy regimen and cycles, pre- and post-neoadjuvant imaging data, surgical procedures, postoperative complications, and pathologic diagnosis and staging. After surgery, patients need to have a CT or MRI radiological evaluation every three months and be followed up with by phone or in-person for a minimum of a year.

Procedures: The neoadjuvant treatment regimen was determined after multidisciplinary discussion and consultation with the patient, in accordance with CSCO guidelines, while comprehensively considering the toxicity spectrum and patient value preferences. All patients completed a minimum of two cycles of neoadjuvant chemoimmunotherapy administered in 21-day intervals. The chemotherapy regimens included three protocols: 1) TP regimen with albumin-bound paclitaxel (260 mg/m²) or paclitaxel (175 mg/m²) combined with cisplatin (75 mg/m²), both delivered via intravenous infusion on day 1; 2) GP regimen utilizing gemcitabine (1000 mg/m²) on days 1 and 8 alongside cisplatin (75 mg/m²) on day 1; and 3) DPF regimen comprising docetaxel (75 mg/m²) and cisplatin (75 mg/m²) on day 1, supplemented by continuous fluorouracil infusion (750 mg/m²) from days 1 to 5. PD-1 inhibitors were administered concurrently, with agents including sintilizumab 200 mg, tislelizumab 200 mg, camrelizumab 200 mg, toripalimab 240 mg, pembrolizumab 200 mg, or serplulimab 200 mg, all given intravenously on day 1 of each cycle. After chemoimmunotherapy, surgery was scheduled 2 to 4 weeks after completion of the last cycle of neoadjuvant therapy. In the event that surgery is not performed more than seven weeks following the final neoadjuvant treatment due to the presence of adverse effects, it may be classified as delayed.

Management of adverse events through standardized processes: Hematological toxicity, such as leukopenia, anemia, thrombocytopenia, symptomatic treatment with G-CSF, blood transfusion, thrombopoietin or dose adjustment; non-hematological events such as nausea and immune enteritis require the use of antiemetic drugs, antidiarrheal drugs, glucocorticoids or discontinuation of PD-1 inhibitors. The multidisciplinary team (MDT) monitored the patients weekly and adjusted the treatment plan according to the CTCAE v 5.0 standard.

Evaluation indicators: The primary outcome was disease-free survival (DFS) and overall survival (OS). The time between the beginning of neoadjuvant therapy and the first recurrence, distant metastasis, or death from any cause was referred to as DFS. OS was defined as either death from any cause during the follow-up or the time between neoadjuvant therapy and the most recent follow-up. Secondary outcomes were radiologic response before surgery, pathological responses including MPR and pathological complete response (pCR), and treatment-related adverse events (TRAEs). The Response Evaluation Criteria in Solid Tumor (RECIST) version 1.1 was used to assess the radiological response. The total of partial response (PR) and complete response (CR) is known as the objective response rate (ORR). MPR is defined as the presence of viable tumor cells in 10% of the initial tumor. The absence of viable tumor cells in the primary tumor and lymph node samples is called pCR. If the proportion of residual viable tumor cells in the primary tumor is > 10%, it is classified as Incomplete Pathological Response (IPR). TRAEs were graded according to the National Cancer Institute’s Common Terminology Criteria for Adverse Events, Version 5.0.

Statistical analysis: DFS and OS were analyzed by the Kaplan-Meier method. Continuous variables were expressed as mean ± standard deviation or median and range, and the Mann-Whitney U test or t test was used for comparison between groups. Categorical variables were expressed as counts and percentages, and the chi-square test or Fisher’s exact test was used for comparison between groups. Log-rank test was used to determine the difference in survival between groups. When *P* < 0.05, the difference was considered statistically significant. All analyses were performed using GraphPad Prism (version 9) and IBM SPSS (version 24).

## 3 Results

Patient characteristics: From January 2019 to May 2024, 82 eligible patients were enrolled in this study. Baseline characteristics are shown in **Table 1**. The median age of patients at the time of diagnosis was 59 years (range 33-75), and most of the patients were males (69, 84.1%). 48.8% (40 patients) had a PS score of 0, and 51.2% (42 patients) had a score of 1, suggesting that the patients had a good overall physical status. Primary tumors were mainly located in the oral cavity (50.0%, n = 41) and oropharynx (24.4%, n = 20), followed by the larynx (14.6%, n = 12) and hypopharynx (11.0%, n = 9). Smoking history was reported in 36.6% (n = 30) of patients, and alcohol consumption history in 57.3% (n = 47). Among oropharyngeal tumors, the P16 positivity rate was 35.0% (n = 7). In terms of tumor staging, the clinical T4 stage was most prevalent (47.6%, n = 39), followed by T2 and T3 stages (both 23.2%), with T1 accounting for 6.1%. For nodal status, 57.3% (n = 47) of patients were in clinical stage N2, 14.6% (n = 12) in N0, 20.7% (n = 17) in N1, and 7.3% (n = 6) in N3. Clinical stage IVA was observed in 64.6% (n = 53) of patients, stage III in 30.5% (n = 25), and stage IVB in 4.9% (n = 4). Extranodal extension (ENE) was detected in 9.8% (n = 8) of cases, whereas 90.2% (n = 74) showed no evidence of such invasion.

**Table 1:** Baseline characteristics (N = 82).

Characteristics	N (%)
Age, median (range), years	59 (range 33-75)
Sex	
Male	69(84.1%)
Female	13(15.9%)
PS scores	
0	40(48.8%)
1	42(51.2%)
Tumor sites	
Oral	41(50.0%)
Oropharyngeal	20(24.4%)
Larynx	12(14.6%)
Hypopharyngeal	9(11.0%)
T category	
T1	5(6.1%)
T2	19(23.2%)
T3	19(23.2%)
T4	39(47.6%)
N category	
N0	12(14.6%)
N1	17(20.7%)
N2	47(57.3%)
N3	6(7.3%)
AJCC stage (the eighth edition)	
III	25(30.5%)
IVA	53(64.6%)
IVB	4(4.9%)
Smoking	
No	30(36.6%)
Yes	52(63.4%)
Drinking	
No	47(57.3%)
Yes	35(42.7%)
P16 status(oropharyngeal)	
Positive	7(35.0%)
Negative	13(65.0%)
ENE	
Positive	8(9.8%)
Negative	74(90.2%)

Neoadjuvant therapy: Detailed characteristics of patient treatment are shown in **Table 2**. All patients in the study received more than two cycles of neoadjuvant chemoimmunotherapy, including albumin, paclitaxel or docetaxel, gemcitabine or fluorouracil, cisplatin, and a PD-1 inhibitor. Specifically, 77 patients (93.9%) received chemotherapy with the TP regimen, while the remaining 3 patients (3.7%) received chemotherapy with the DPF regimen and 2 patients (2.4%) received chemotherapy with the GP regimen. Among the PD-1 inhibitors, sintilimab was used in 39 patients (47.6%), and pembrolizumab in 17 patients (20.7%), being the most commonly applied agents. In terms of treatment cycles, 32 patients (39.0%) received 2 cycles of neoadjuvant therapy, whereas 50 patients (61.0%) underwent 3 or more cycles.

**Table 2:** Treatment-related characteristics (N=82).

Characteristics	Patients (n = 82)
Chemotherapy	
TP	77(93.9%)
DPF	3(3.7%)
GP	2(2.4%)
Immunotherapy regimen, No. (%)	
Sintilimab	39(47.6%)
Tirelizumab	7(8.5%)
Camrelizumab	3(3.7%)
Toripalimab	13(15.8%)
Pembrolizumab	17(20.7%)
Serplulimab	3(3.7%)
Neoadjuvant cycle, No. (%)	
2	32(39.0%)
3	41(50.0%)
4	8(9.8%)
5	1(1.2%)

Surgical treatment: The surgical operation details are presented in **Table 3**. The median interval from neoadjuvant chemoimmunotherapy to surgery was 25 days (range: 12–59 days). One patient (1.2%) had delayed surgery due to COVID-19. The median blood loss was 200 ml (range: 20-900ml), and the median hospital stay was 17 days (range: 5-31days). Intraoperatively, four patients had positive margins, while 78 patients (95.1%) achieved R0 resection. Eleven patients developed postoperative complications, which included flap tip necrosis, pharyngeal fistula, infection, and operative site bleeding. Notably, no patient died or suffered from other severe surgical complications within 30 or 90 days post-surgery.

**Table 3:** Surgical procedures(N=82).

Characteristics	Patients (n = 82)
The time interval between the date of surgery and the last neoadjuvant therapy, median (range), days	25(12-59)
Bleeding, median (range) mL	200(20-900)
Hospitalization time, median (range), days	17(5-31)
Resection margins	
R0	78(95.1%)
R1	4(4.9%)
R2	0
Surgical complication	
Necrosis of the end of the flap	2(2.4%)
Pharyngeal fistula	1(1.2%)
Infection	5(6.1%)
Bleeding in the operated area	3(3.6%)
Postoperative risk factors	
Multiple lymph node metastases in the neck (≥2)	52(63.4%)
Vessel carcinoma embolus	6(7.3%)
Perineural invasion	5(6.1%)
Unclean excision margin	4(4.9%)
Extranodal extension, ENE	8(9.8%)

**Figure 1:**
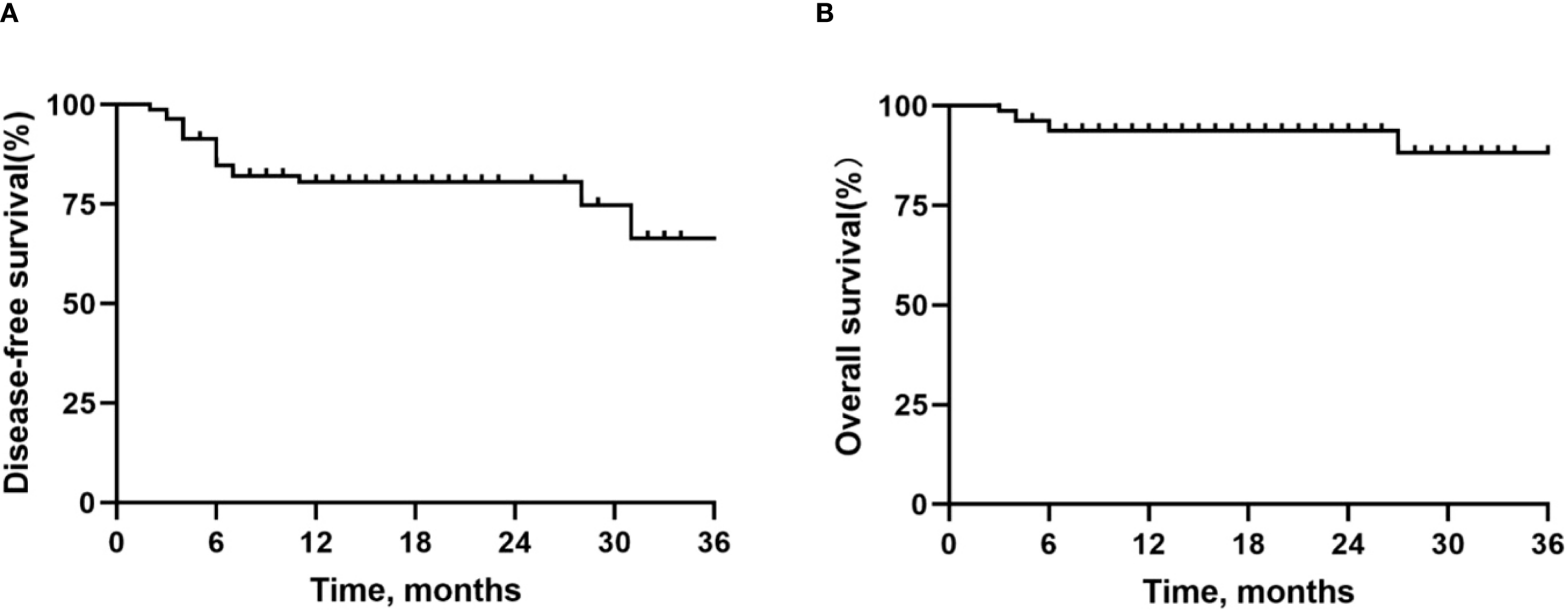
Kaplan-Meier survival curves in all patients. **(A)** Disease-free survival. **(B)** Overall survival.

Outcomes: **Table 4** outlines the radiological and pathological responses. Imaging assessment showed that 5 patients (6.0%) achieved CR, 60 patients (73.2%) achieved PR, and 17 patients (20.7%) had stable disease (SD), with an ORR of 79.3%. Postoperative pathological analysis showed that 34 patients (41.5%) achieved MPR, 21 patients (25.6%) achieved pCR, and 48 patients (58.5%) had IPR. As of the data cutoff date on 1 September 2024, following a median follow-up of 16 months (range: 3–48 months), 76 out of 82 patients (92.7%) remained alive, and 65 patients (79.3%) were free from recurrence. Disease progression was observed in 17 patients (20.7%) who underwent surgery, of whom 6 (7.3%) succumbed to the disease. The median DFS and OS were not reached for the entire patient cohort. The 12-month, 24-month, and 36-month DFS rates were 80.5%, 80.5%, and 66.5%, respectively; the corresponding OS rates were 93.7%, 93.7%, and 88.2%. For detailed visual representation, refer to **Figure 1**.

**Table 4:** Radiologic and pathologic responses.

Characteristics	Patients (n = 82)
Radiologic responses	
Complete response	5
Partial response	60
Stable disease	17
Pathologic responses	
Incomplete pathological response	48
Major pathological response	34
Complete pathological response	21

Safety assessment: TRAEs during neoadjuvant therapy are detailed in **Table 5**. All patients experienced TRAEs of varying severity during neoadjuvant therapy that did not result in discontinuation of the overall study treatment, delayed surgery, or death. The overall incidence of treatment-related adverse events was 93.9% (77/82), with a grade 3/4 incidence of 14.6% (12/82) and no grade 5 adverse event. The most common TRAEs were leukopenia (69.5%, 57/82), anemia (65.9%, 54/82), and nausea and vomiting (45.1%, 37/82). Grade 3/4 TRAEs included leukopenia (6.1%, 5/82), anemia (4.9%, 4/82), thrombocytopenia (3.7%, 3/82), and transaminase elevation (1.2%, 1/82) and immune enteritis (1.2%, 1/82).

**Table 5:** Treatment-related adverse events during neoadjuvant treatment (n = 82).

Adverse events	Grade 1–2	Grade 3	Grade 4
Nausea and vomiting	37(45.1%)	0	0
Fatigue	15(18.3%)	0	0
Mucositis	15(18.3%)	8(9.8%)	0
Leucopenia	57(69.5%)	3(3.7%)	2(2.4%)
Anemic	54(65.9%)	3(3.7%)	1(1.2%)
Thrombocytopenia	8(9.8%)	2(2.4%)	1(1.2%)
Rashes	4(4.9%)	1(1.2%)	0
Transaminase elevation	12(14.6%)	1(1.2%)	0
Elevated creatinine	6(7.3%)	0	0
Hypothyroidism	11(13.4%)	0	0
Immunomyocarditis	2(2.4%)	0	0
Immune enteritis	0	1(1.2%)	0
Myalgia/Arthralgia	4(4.9%)	0	0

Factors associated with pathological reactions: In this study, the correlation between patient characteristics and pathological response was further explored to identify a group of patients likely to achieve a favorable pathological response. Statistical analysis revealed that several factors, including gender, age, history of smoking, history of alcohol consumption, pre-treatment clinical stage, chemotherapy regimen, type of PD-1, the interval between the end of neoadjuvant therapy and surgery, neoadjuvant therapy cycles and the presence of ENE, did not show statistically significant differences between patients in the MPR group and the IPR group (*P*>0.05, **Table 6**). However, when evaluating the baseline characteristics of all patients, a positive correlation was found between the primary site of the tumor and the pathological response (*P* = 0.026, **Table 6**). Further pairwise comparative analysis revealed that patients with HPV- positive oropharyngeal cancer exhibit a more favorable pathological response compared to HPV- negative patients (2). However, in our study cohort, there were still three HPV-negative patients who achieved MPR or PCR and one HPV-positive oropharyngeal cancer patient who achieved IPR, suggesting that HPV infection is not the sole determinant of pathological response to neoadjuvant treatment of oropharyngeal cancer. In addition, patients’ PS scores showed a significant positive correlation with pathologic response (*P* < 0.001, **Table 6**). Specifically, patients with lower preoperative PS scores typically retained more intact immune function and more adequate immune responses, resulting in better treatment outcomes. Additionally, this investigation discovered that nearly all postoperative problems happened to IPR patients (10/11; *P* = 0.011, **Table 6**). Therefore, more rigorous and comprehensive preoperative surgical evaluation and planning should be performed in patients with potentially suboptimal outcomes.

**Table 6:** Correlation analysis between clinical characteristics and pathological responses (N= 82).

Characteristics	MPR group	IPR group	χ^2^/t	P value
Age	57.0 (53.0, 62.0)	60.0 (52.0, 65.5)	-1.444	0.149
Sex				
Male	27	42	0.976	0.323
Female	7	6		
PS scores				
0	26	14	17.825	**<0.001**
1	8	34		
Tumor sites				
Oral	19	22	10.684	**0.026**
Oropharynx, HPV^+^	6	1		
Oropharynx, HPV^-^	2	11		
Larynx	5	7		
Hypopharyngeal	2	7		
T category				
T1	2	3	0.419	0.969
T2	9	11		
T3	7	12		
T4	16	22		
N category				
N0	6	6	3.272	0.352
N1	8	9		
N2	16	31		
N3	4	2		
AJCC stage (the eighth edition)				
III	13	12	4.126	0.109
IVA	18	35		
IVB	3	1		
Smoking				
No	14	16	0.528	0.468
Yes	20	32		
Drinking				
No	20	27	0.054	0.816
Yes	14	21		
ENE				
Positive	3	5	0.058	0.810
Negative	31	43		
Chemotherapy				
TP	30	45	1.202	0.698
DPF	2	2		
GP	2	1		
Immunotherapy regimen, No. (%)				
Sintilimab	17	22	2.841	0.784
Tirelizumab	2	5		
Camrelizumab	1	2		
Toripalimab	6	7		
Pembrolizumab	8	9		
Serplulimab	0	3		
Neoadjuvant cycles				
2	13	19	0.015	0.902
3 or more	21	29		
Postoperative complication				
No	33	38	6.498	**0.011**
Yes	1	10		

Bold values indicate a statistically significant difference (p< 0.05).

**Figure 2:**
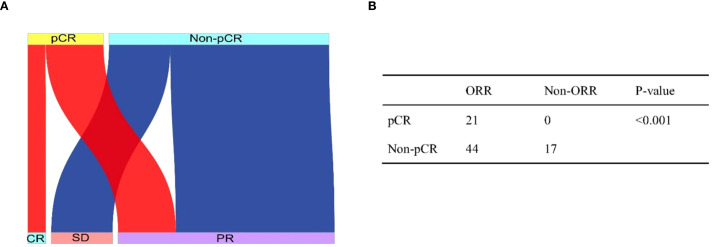
Sankey plot shows the relationship between radiographic response and pathologic response **(A)**. The consistency between radiographic response and pathologic response analyzed by using the two-sided Fisher exact test **(B)**.

Consistency evaluation of imaging and pathological responses: In 82 patients, according to RESIST 1.1, all patients who achieved CR also achieved MPR, while no patients with SD achieved pCR or MPR. Specifically, 5 patients with pCR demonstrated CR, and 16 patients with pCR showed PR (**Figure 2A**). Statistical analysis revealed that patients who achieved ORR were more likely to achieve pCR (*P* < 0.001, **Figure 2B**), indicating a certain consistency between imaging evaluation and pathological evaluation.

### 3.1 Subgroup analysis

**Figure 3:**
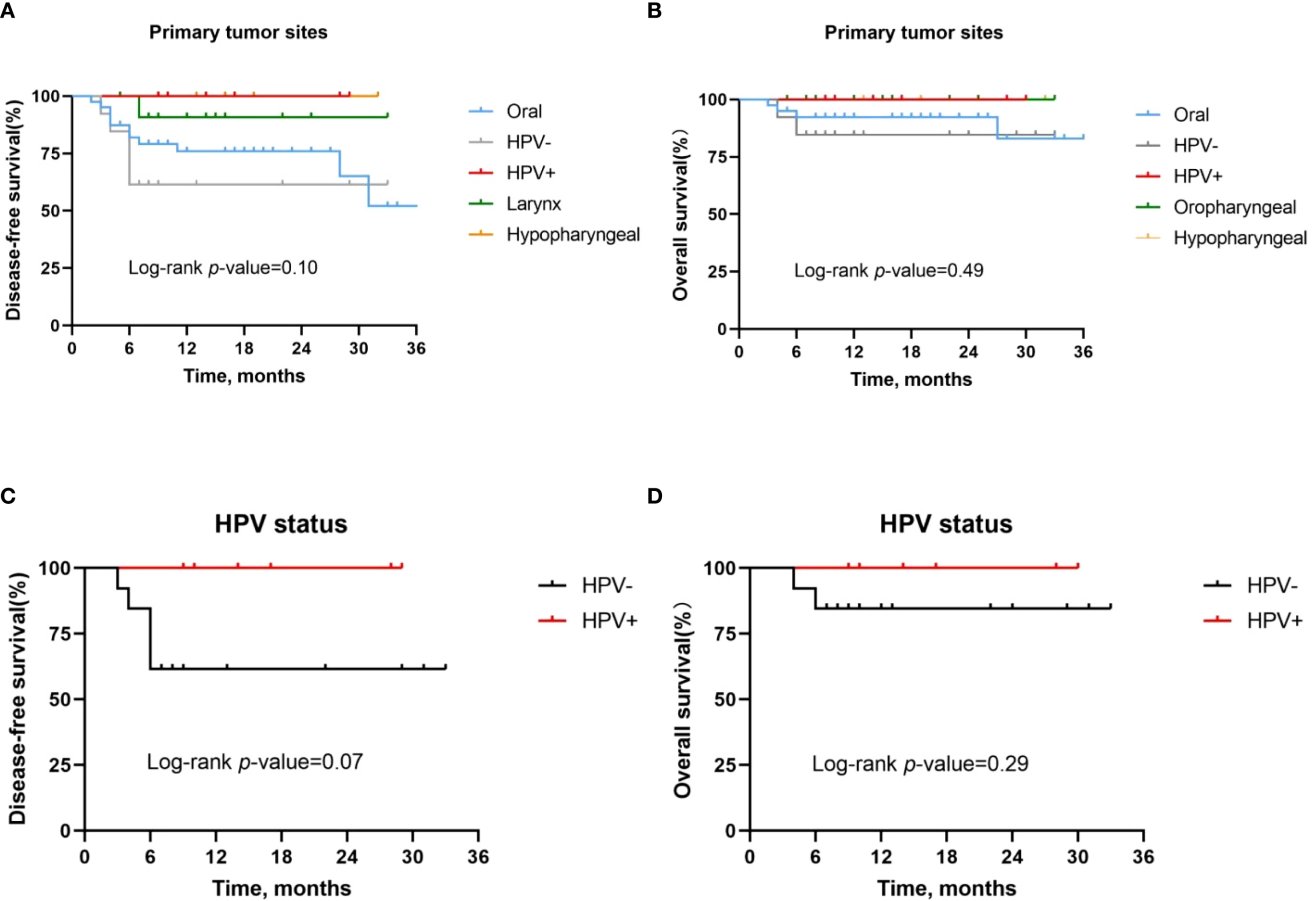
Kaplan-Meier survival curves stratified by tumor site and HPV status. **(A, B)** Disease-free survival **(A)** and overall survival **(B)** for all patients stratified by tumor site. **(C, D)** Disease-free survival **(C)** and overall survival **(D)** for all patients stratified by HPV status.

Then we analyzed the risk factors that might affect the survival time of HNSCC patients treated with neoadjuvant chemoimmunotherapy. First, primary tumor site and HPV infection status were initially investigated for their predictive value regarding OS and DFS. As previously reported, patients with HPV-positive oropharyngeal cancer had a better pathologic response compared with HPV-negative patients, achieving 1-year and 2-year DFS and OS rates of 100%. However, the DFS rate (*P* = 0.10, **Figure 3A**) and OS rate (*P* = 0.49, **Figure 3B**) of HNSCC patients with different primary tumors did not show significant differences. Further analysis showed that there was no significant difference in DFS rate (*P* = 0.07, **Figure 3C**) and OS rate (*P* = 0.29, **Figure 3D**) between HPV-positive and HPV-negative oropharyngeal cancer patients.

**Figure 4:**
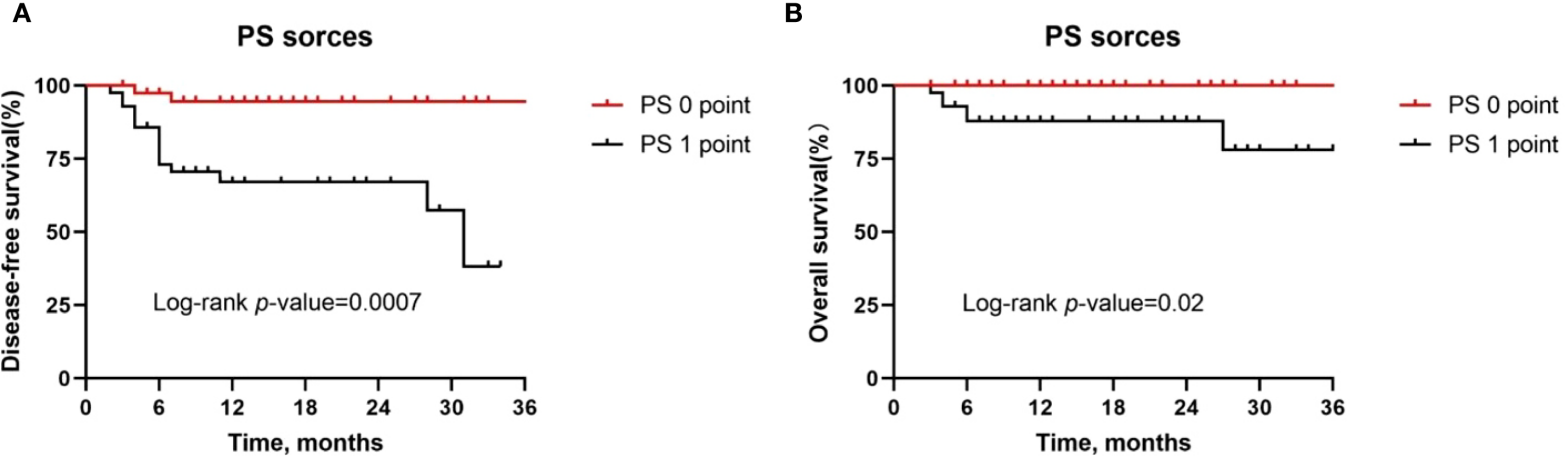
Kaplan-Meier survival curves stratified by PS score. **(A, B)** Disease-free survival **(A)** and overall survival **(B)** for all patients by tumor site.

Second, patients with a PS score of 0 had significantly longer DFS (*P* = 0.0007, **Figure 4A**) and OS (*P* = 0.02, **Figure 4B**) compared to patients with a PS score of 1. Therefore, for patients with poor physical status, we recommend multidisciplinary assessment before treatment and optimization of supportive therapy to improve tolerability.

**Figure 5:**
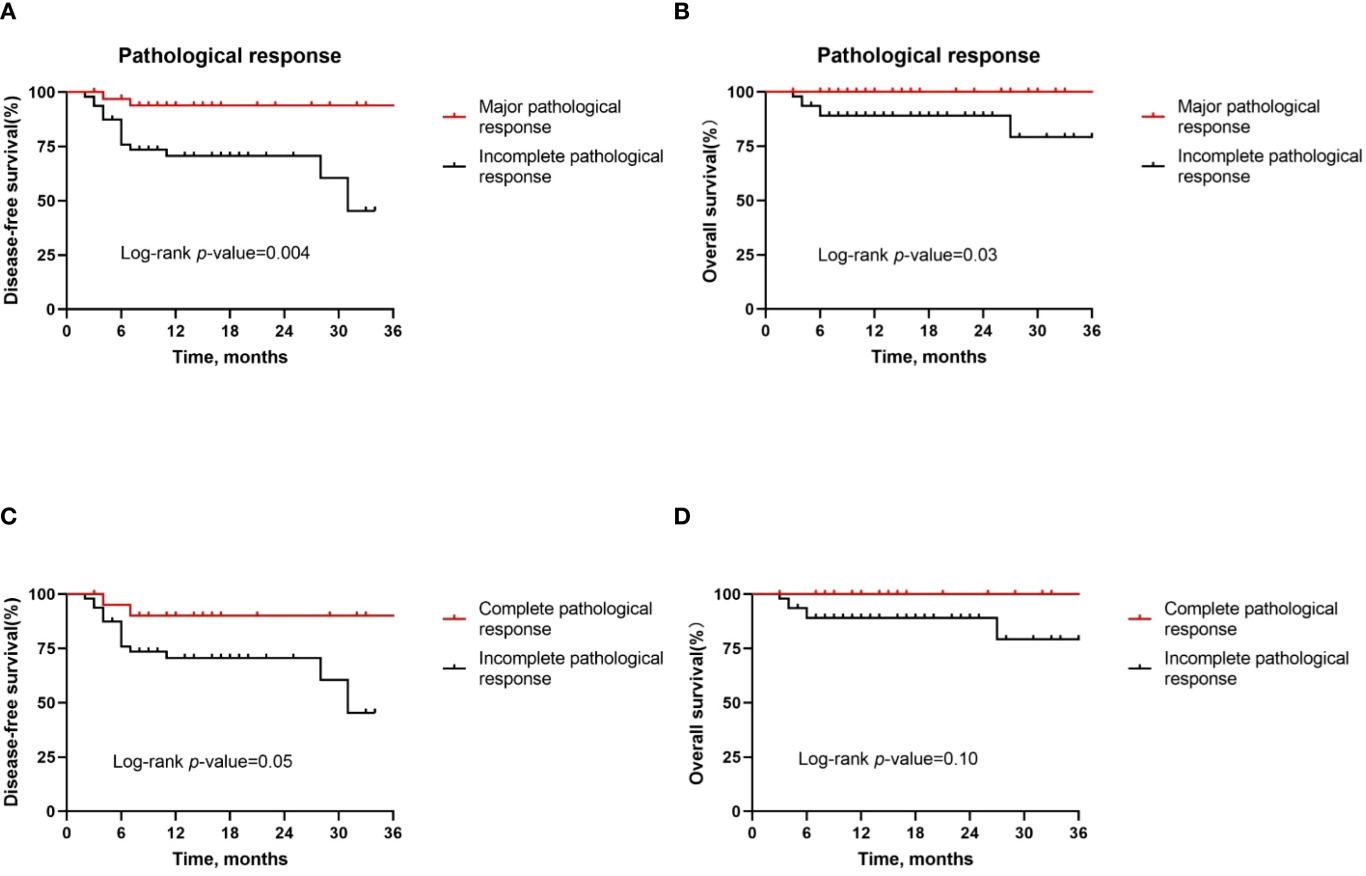
Kaplan–Meier survival curves stratified by pathological responses. **(A, B)** Disease-free survival **(A)** and overall survival **(B)** for all patients by major pathological response. **(C, D)** Disease-free survival **(C)** and overall survival **(D)** for all patients by complete pathological response.

Next, in a subgroup analysis of patients with different pathologic responses, we found that MPR patients had better DFS rates (*P* = 0.004, **Figure 5A**) and OS rates (*P* = 0.03, **Figure 5B**) than IPR patients. However, the DFS rate (*P* = 0.05, **Figure 5C**) and OS rate (*P* = 0.10, **Figure 5D**) were not statistically significant between pCR patients and IPR patients.

**Figure 6:**
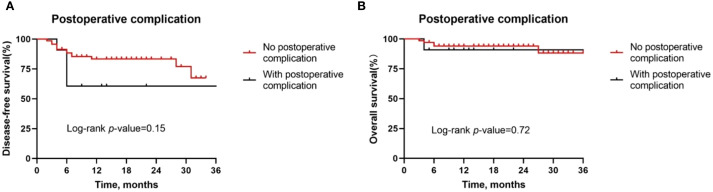
Kaplan-Meier survival curves stratified by the presence of postoperative complications. **(A, B)** Disease-free survival **(A)** and overall survival **(B)** for all patients by tumor site.

Then, our study also found that the 12 patients with a history of head and neck radiotherapy had a significantly lower DFS rate (p=0.0014, **Figure 6A**) and OS rate (p<0.0001, **Figure 6B**) compared to those without a history of radiotherapy, and the difference in survival between the two groups was statistically significant.

**Figure 7:**
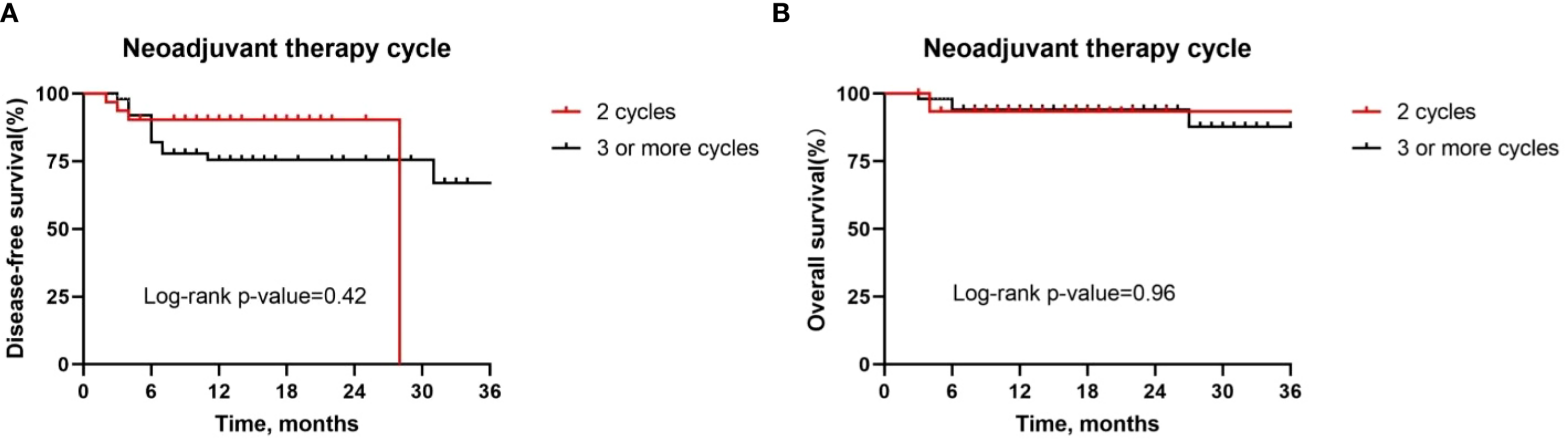
Kaplan-Meier survival curves stratified by the presence of postoperative complications. **(A, B)** Disease-free survival **(A)** and overall survival **(B)** for all patients by tumor site.

**Figure 8:**
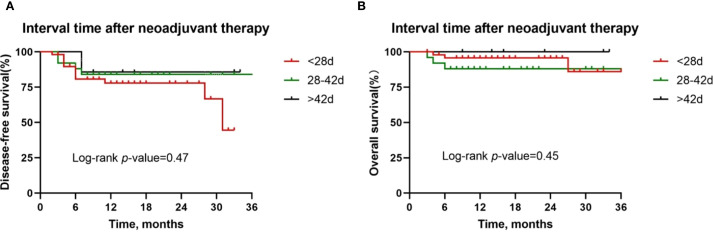
Kaplan-Meier survival curves stratified by time between neoadjuvant last treatment and surgery. A and B Disease-free survival **(A)** and overall survival **(B)** for all patients by tumor site.

Besides, although postoperative complications were found to be positively correlated with pathologic response, there was no difference in the DFS rate (*P* = 0.15, **Figure 7A**) and OS rate (*P* = 0.72, **Figure 7B**) between patients with or without postoperative complications. Similarly, we also observed similar DFS curves (*P* = 0.42, **Figure 8A**) and OS (*P* = 0.96, **Figure 8B**) curves between patients receiving two neoadjuvant therapy cycles versus those with three or more cycles, although, as noted above, an increase in neoadjuvant therapy cycles would be expected to improve the pathologic response. Therefore, imaging evaluations should be performed after two cycles of neoadjuvant therapy to decide whether to continue treatment. If the patient achieves a partial response or better, additional cycles of neoadjuvant therapy may be considered.

Furthermore, we conducted exploratory analyses to identify potential risk factors influencing survival outcomes in patients with locally advanced HNSCC treated with neoadjuvant chemoimmunotherapy. Given the limited follow-up duration, our survival analysis focused primarily on DFS. In the univariate analysis (**Table 7**), PS score (HR = 2.34; 95% CI 1.28–4.27; *P* = 0.005), history of prior head and neck radiotherapy (HR = 2.61; 95% CI 1.35–5.03; *P* = 0.004), and pathological response (HR = 0.42; 95% CI 0.20–0.87; *P* = 0.014) were significantly associated with DFS. Multivariate analysis further identified prior head and neck radiotherapy history (HR = 2.89; 95% CI 1.03–8.11; p= 0.044) as an independent predictor of worse DFS (**Table 7**).

**Table 7:** Univariate and multivariate analyses of risk factors for disease-free survival.

Variables	Univariate		Multivariate	
	HR (95% CI)	P value	HR (95% CI)	P value
PS scores		0.005		0.062
0	1.000		1.000	
1	8.337 (1.900, 36.589)	0.005	4.552 (0.925, 22.416)	0.062
History of radiation in Head and neck		0.004		0.044
No	1.000			
Yes	4.433 (1.615, 12.171)	0.004	2.887 (1.027, 8.114)	0.044
Pathological response		0.014		0.248
MPR	1.000			
IPR	6.376 (1.455, 27.933)	0.014	2.593 (0.514, 13.085)	0.248

## 4 Discussion

This is an observational study of patients with locally advanced HNSCC to investigate the efficacy and safety of surgery after neoadjuvant chemoimmunotherapy. The study demonstrated that this neoadjuvant treatment significantly improved the rate of pathological response and that patients achieved good survival, as well as the safety and tolerability of the treatment. In terms of safety, the incidence of grade 3/4 TRAEs was 14.6% (12/82), which was significantly lower than the previous incidence of 21.7% (16) and 37% (21) reported in the literature, and most of the TRAEs were chemotherapy-related. In addition, none of the patients in this cohort experienced disease progression during neoadjuvant therapy, suggesting that this regimen did not increase the risk of uncontrolled tumors.

Although neoadjuvant chemoimmunotherapy has not yet been included in the standard treatment of locally advanced HNSCC, its significant clinical value has been supported by data from several studies. With the publication of the results of studies such as CheckMate-141 and KEYNOTE-048, immunotherapy has successfully ranked among the first-line treatment options for patients with recurrent or metastatic HNSCC (22, 23). Among them, the KEYNOTE-048 study confirmed that pembrolizumab in combination with chemotherapy significantly improved OS in patients with relapsed or metastatic HNSCC compared with the conventional EXTREME regimen (23), which provided a solid theoretical basis for the neoadjuvant immunotherapy strategy for locally advanced HNSCC. Studies have shown that neoadjuvant PD-1 inhibitors alone for locally advanced HNSCC have relatively low MPR and pCR rates, whereas combining them with other therapies significantly improves these key efficacy metrics (12, 13, 24). Available clinical trial data show that neoadjuvant chemoimmunotherapy has a pCR rate ranging from 16.7%-55.6%, an MPR rate ranging from 27.8%-81.58%, and a 1-year DFS of more than 85%, which fully confirms the significant efficacy of this regimen in the treatment of locally advanced HNSCC (16, 17). In our study cohort, we conducted a systematic analysis of 82 patients with locally advanced HNSCC who received neoadjuvant chemoimmunotherapy. The results showed that 25.6% and 41.5% of patients achieved pCR and MPR, respectively. After a median follow-up of 16 months, the 1-year DFS and OS rates reached 80.5% and 97.4%, respectively, further validating the clinical value of neoadjuvant chemoimmunotherapy in the treatment of locally advanced HNSCC. These data provide an important reference basis for conducting larger prospective clinical studies in the future.

The present study also observed an ORR as high as 79.3% after neoadjuvant therapy, indicating a significant synergistic effect of immunotherapy combined with chemotherapy, which can bring about better tumor response. Several studies have reported that neoadjuvant chemoimmunotherapy regimens for locally advanced HNSCC can significantly enhance the ORR rate of patients and bring better survival (15, 19, 25). Furthermore, complete surgical resection is always a key component of radical treatment in clinical decision-making for locally advanced HNSCC (4, 26). The high ORR resulting from neoadjuvant therapy has three clinical values: first, by rapidly reducing the volume of the primary focus and improving the anatomical relationship between the tumor and the peripheral neurovascular vessels, it converts cases initially assessed as unresectable to operable status (27–29); secondly, fibrotic changes in the tumor border after treatment can improve the accuracy of intraoperative margin determination, and relevant studies have shown that the R0 resection rate of patients after neoadjuvant combination therapy has been significantly increased; thirdly, for lesions involving laryngeal, oropharyngeal, hypopharyngeal, and other functionally preserved areas, the reduction of tumor volume can effectively increase the rate of laryngeal preservation without affecting the efficacy of treatment, and significantly improve the quality of life of patients (30–33).

The correlation between pathological response and imaging response after neoadjuvant chemoimmunotherapy is currently inconclusive. In the present study, we found a significant correlation between imaging response and pathological response, with patients who achieved ORR on imaging evaluation after neoadjuvant therapy being more likely to achieve pCR, consistent with similar previous results with neoadjuvant single-agent immunotherapy or immune-combination therapy (34, 35). However, it has also been shown that conventional imaging assessments have significant limitations in reflecting pathological response, often underestimating the actual degree of pathological response (14, 36). Based on this finding, the clinical value of single reliance on imaging metrics for pathological response prediction has been questioned. In the future, further expansion of the sample size is needed to construct a multifactorial integrated column-line diagram to predict the incidence of MPR.

As we know, HPV infection has become an important causative factor for HNSCC (2, 37, 38). HPV-positive oropharyngeal cancer has a relatively good prognosis because it is more sensitive to radiotherapy and immune checkpoint blockade therapy due to its unique clinical and pathological features (39–42). In this study, HPV-positive oropharyngeal cancer showed the best pathological response and survival benefit to neoadjuvant chemoimmunotherapy, which is consistent with the findings of the KEYNOTE-012 study (43) and the Checkmate 141 study (42). In addition, the CheckMate 358 trial evaluated the safety and feasibility of neoadjuvant treatment with nivolumab monotherapy in patients with resectable HNSCC. The results showed that patients in the HPV-positive group had an MPR of 5.9% (12), which was significantly lower than the 41.5% in this study. This difference suggests that the combination of immunotherapy and chemotherapy may reverse the immunosuppressive microenvironment of HPV-negative tumors through a synergistic effect. Further analyses suggest that the highly immunogenic tumor microenvironment associated with HPV infection may be a key factor contributing to the limited efficacy of single-agent immunotherapy, which is overcome by the combination regimen or by the enhancement of the immune response (44, 45).

The present study also found that patients with PS score 0 had significantly better DFS and OS than patients with PS score 1, and that patients with PS score 0 were more likely to achieve MPR. This result is consistent with previous studies, which have shown that patients with lower PS scores usually have better physiological status and immune function, and can activate anti-tumor immune responses more effectively (46, 47). In addition, patients with a PS score of 0 have higher treatment adherence and may consequently experience more significant pathological response and survival benefit (48). For patients with poorer physical status, multidisciplinary assessment before treatment is recommended to optimize supportive therapy to improve tolerability.

The duration of cycles of neoadjuvant chemoimmunotherapy should also be included in the discussion. Huang (16) et al. noted that in a two-cycle regimen using gemcitabine and cisplatin in combination with toripalimab, the pCR and MPR rates were 16.7% and 27.8%, respectively, which were significantly lower than in other similar clinical trials. This phenomenon may be attributed to a dual mechanism: two cycles of neoadjuvant therapy may not be sufficient to fully activate the systemic anti-tumor immune response; at the same time, the tumor regression effect of gemcitabine on HNSCC was weaker than that of paclitaxel-based chemotherapeutic agents, limiting the synergistic efficacy of the immunotherapy. Uppaluri (24) et al. further validated that compared with the single-cycle regimen, two cycles of neoadjuvant immunotherapy can improve the pathological response rate. This suggests that increasing the number of treatment cycles may have a positive effect on enhancing efficacy. However, prolonging the duration of neoadjuvant chemoimmunotherapy may increase the risk of disease progression or hyperprogression in patients with poorer treatment response, a point that emphasizes the importance of close monitoring during treatment. In our cohort, patients receiving three cycles of neoadjuvant therapy were found to achieve a better pathological response. Nevertheless, we did not observe a positive effect of additional treatment cycles on survival, suggesting that IPR patients may face disease progression during neoadjuvant therapy. Also, IPR patients showed a higher rate of postoperative complications, which could be attributed to increased surgical risk due to tumor progression during neoadjuvant therapy. In light of this, we recommend imaging evaluation after two cycles of neoadjuvant chemoimmunotherapy to decide whether to continue treatment. If the patient achieves a partial response or better, additional cycles of neoadjuvant therapy may be considered.

Concerns about neoadjuvant therapy in previous studies have centered on the risk of disease progression during treatment and the possibility of surgical delay (49). However, no disease progression occurred during neoadjuvant therapy in our patients. Of the 82 patients who participated in the study, 53 (64.6%) had N2–3 disease, traditionally considered a high risk for surgical resection. With neoadjuvant chemoimmunotherapy, 62.2% (51/82) of the patients achieved clinicopathological downstaging, resulting in the conversion of an otherwise large tumor from unresectable to resectable, significantly improving the R0 resection rate. In addition, neoadjuvant therapy not only rapidly reduced the tumor size but also narrowed the surgical scope and improved short-term organ preservation without increasing intraoperative risk. There were no serious complications within 3 months after surgery, confirming that this regimen combines both efficacy and safety.

Our study has several limitations. First, as a single-arm retrospective analysis, it is inherently subject to selection bias in patient screening and treatment allocation; therefore, the findings require validation in prospective randomized controlled trials. Second, the limited sample size may compromise the generalizability and reliability of the results. In the future, the clinical data of different treatment strategies will be systematically collected through a combination of multicenter real-world data collection and phase III clinical trials to more comprehensively verify the clinical benefits of neoadjuvant chemoimmunotherapy. In addition, the median follow-up period of 16 months is not sufficient to adequately assess the risk of distant relapse and delayed toxicity and to fully establish the long-term survival benefit of neoadjuvant therapy. Notably, most published clinical trials related to neoadjuvant chemotherapy or immunotherapy for squamous head and neck cancer have not reached the observational time window for reporting long-term survival endpoints. Given this, we will continue to follow up the study cohort and plan to further define the long-term impact of neoadjuvant chemoimmunotherapy on survival outcomes through systematic analyses of 3- and 5-year survival data.

In conclusion, our study demonstrated that neoadjuvant chemoimmunotherapy followed by surgery significantly improved pathological response rates, R0 resection rates, and survival outcomes in patients with locally advanced HNSCC, while simultaneously reducing the incidence of severe TRAEs. Notably, enhanced pathological responses were observed in HPV-positive patients with a PS score of 0. Consequently, prospective clinical trials with larger sample sizes are needed to further validate these findings and to use this approach as a potential therapeutic strategy for the management of locally advanced HNSCC.

## Data Availability

The original contributions presented in the study are included in the article/Supplementary Material. Further inquiries can be directed to the corresponding authors.
